# Radiolabeled Bombesin Analogs

**DOI:** 10.3390/cancers13225766

**Published:** 2021-11-17

**Authors:** Rosalba Mansi, Berthold A. Nock, Simone U. Dalm, Martijn B. Busstra, Wytske M. van Weerden, Theodosia Maina

**Affiliations:** 1 Division of Radiopharmaceutical Chemistry, Clinic of Radiology and Nuclear Medicine University Hospital Basel, 4031 Basel, Switzerland; rosalba.mansi@usb.ch; 2 Molecular Radiopharmacy, INRaSTES, NCSR “Demokritos”, 15310 Athens, Greece; nock_berthold.a@hotmail.com; 3 Erasmus Medical Center Rotterdam, 3000 CA Rotterdam, The Netherlands; s.dalm@erasmusmc.nl (S.U.D.); m.busstra@erasmusmc.nl (M.B.B.); w.vanweerden@erasmusmc.nl (W.M.v.W.)

**Keywords:** gastrin-releasing peptide receptor, radiolabeled bombesin, cancer theranostics

## Abstract

**Simple Summary:**

Recent medical advancements have strived for a personalized medicine approach to patients, aimed at optimizing therapy outcomes with minimum toxicity. In this respect, nuclear medicine methodologies have been playing increasingly important roles. For example, the overexpression of peptide receptors, such as the gastrin-releasing peptide receptor (GRPR), on tumor cells as opposed to their lack of expression in healthy surrounding tissues can be elegantly exploited with the aid of “smart” peptide carriers, such as the analogs of the amphibian 14-peptide bombesin (BBN). These molecules can bring clinically attractive radionuclides to malignant lesions in prostate, breast, and other human cancers, sparing healthy tissues. Depending upon the radionuclide in question, diagnostic imaging with single-photon emission computed tomography (SPECT) or positron emission tomography (PET) has been pursued, identifying patients who are eligible for peptide radionuclide receptor therapy (PRRT) in an integrated “theranostic” approach. In the present review, we (i) discuss the major steps taken in the development of anti-GRPR theranostic radioligands, with a focus on those selected for clinical testing; (ii) comment on the present status in this field of research; and (iii) reflect on the current limitations as well as on new opportunities for their broader and more successful clinical applications.

**Abstract:**

The gastrin-releasing peptide receptor (GRPR) is expressed in high numbers in a variety of human tumors, including the frequently occurring prostate and breast cancers, and therefore provides the rationale for directing diagnostic or therapeutic radionuclides on cancer lesions after administration of anti-GRPR peptide analogs. This concept has been initially explored with analogs of the frog 14-peptide bombesin, suitably modified at the N-terminus with a number of radiometal chelates. Radiotracers that were selected for clinical testing revealed inherent problems associated with these GRPR agonists, related to low metabolic stability, unfavorable abdominal accumulation, and adverse effects. A shift toward GRPR antagonists soon followed, with safer analogs becoming available, whereby, metabolic stability and background clearance issues were gradually improved. Clinical testing of three main major antagonist types led to promising outcomes, but at the same time brought to light several limitations of this concept, partly related to the variation of GRPR expression levels across cancer types, stages, previous treatments, and other factors. Currently, these parameters are being rigorously addressed by cell biologists, chemists, nuclear medicine physicians, and other discipline practitioners in a common effort to make available more effective and safe state-of-the-art molecular tools to combat GRPR-positive tumors. In the present review, we present the background, current status, and future perspectives of this endeavor.

## 1. Introduction

**Figure 1:**
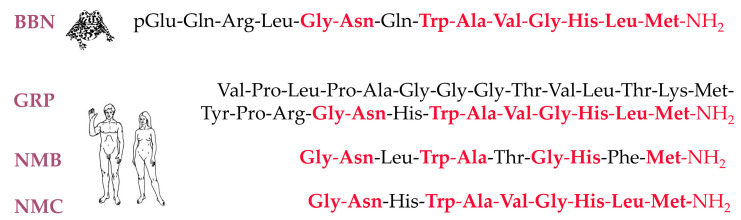
Amino acid sequence of frog BBN and the respective GRP, NMC, and NMB peptides, native in humans; conserved residues are indicated by red color.

The latest trends in medicine involve a personalized approach to patients. Such a patient-tailored approach can be well served by nuclear medicine techniques in oncological patients, following the so-called “theranostic” concept. This concept can be well exemplified in the case of bombesin and its analogs. Bombesin (BBN) is a 14-peptide (Figure 1) first isolated from the skin of the European fire-bellied toad *Bombina bombina* and exerting a series of actions on mammal tissues through binding to distinct G-protein coupled receptors (GPCRs), known as bombesin receptors (BB-Rs) [1,2]. Interestingly, a number of BBN-like peptides have been identified in humans: the 27-mer gastrin-releasing peptide (GRP) and two other C-terminal decapeptide versions, namely neuromedin Β (NMΒ) and neuromedin C (NMC, GRP(18–7)), all with a high sequence homology (Figure 1). The bombesin receptor family comprises three receptor subtypes in mammals, which are pharmacologically distinguished by their distinct affinities to native peptides. BB_1_-R, also known as NMBR, shows high affinity for NMB; BB_2_-R, also known as GRPR, strongly binds GRP and NMC; BB_3_-R is an orphan receptor with no native ligand identified thus far; BBN shows high binding affinities for both NMBR and GRPR, but not for BB_3_-R. 

The GRPR, in particular, has attracted much attention in oncology and in nuclear medicine by virtue of its high-density expression in major human cancers, such as prostate [3,4,5], breast [6,7,8,9], and small-cell lung cancer [10], as well as in gastrinoma, gastrointestinal stromal tumors [11,12], and other cancer types [13]. This finding in combination with the lack of physiological GRPR expression in healthy tissues (with the exception of the GRPR-rich pancreas and gastrointestinal tract [14]) has provided the rationale for using properly designed BBN-like peptide carriers of radionuclides in malignant lesions for cancer theranostics [2,15,16]. BBN and/or GRP, but mostly their C-terminal fragments that still retain affinity for the GRPR, have been employed as motifs for anti-GRPR radioligand development. For such a purpose, suitable radiometal-binding chelators are covalently attached in their N-terminus, either directly or via a linker, and “theranostic radiopeptide pairs” may become available. Accordingly, the peptide conjugate is used for labeling with diagnostic gamma emitting radiometals (e.g., Tc-99m and In-111) for single-photon emission computed tomography (SPECT), or a positron emitter (e.g., Ga-68 and Cu-64) for positron emitter tomography (PET) imaging. The imaging will elucidate disease spread and monitor disease progress as well as post-therapy responses. Moreover, the imaging will identify patients eligible for peptide receptor radionuclide therapy (PRRT), using the same peptide conjugate labeled, instead, with a particle emitter, either a beta (e.g., Lu-177 and Y-90) or an alpha (e.g., Ac-225 and Bi-213) emitter. The concept of combining diagnostic and therapeutic tools in the same vector has already been successfully applied in nuclear medicine in the case of somatostatin and represents a valid example of personalized and precision medicine [17,18].

In the present review, we briefly discuss the development of GRPR radioligands, from its early days to its present status, with an emphasis on analogs involved in translational studies in breast and prostate cancer. Keeping a strong focus on theranostics of GRPR-positive cancer, we limit this review to peptide analogs labeled with diagnostic or therapeutic radiometals, excluding the F-18 analogs suitable for PET imaging only. Likewise, delivery platforms loaded with BBN-like domains, such as nanoparticles, are not included in this review. The shift from GRPR agonists to antagonists is more thoroughly discussed, in view of the plethora of preclinical and clinical studies on this topic. After briefly outlining the issue of metabolic stability, the new trends in GRPR-targeted cancer theranostics are described, revealing the challenges and new opportunities in the field. Of particular interest is the final part of this review, in which we present the views of a clinician on the limitations, strengths, and future opportunities in prostate cancer theranostics using GRPR-targeted radiolabeled bombesin analogs. 

## 2. First BBN-like Radioligands in Nuclear Oncology: GRPR Agonists

Most efforts involving BBN-like radioligands have been initially directed to the diagnosis and treatment of prostate cancer, a major cause of death worldwide [19]. While GRPR is highly expressed in prostatic intraepithelial neoplasia, as well as primary and invasive prostatic carcinoma, it is practically absent in normal prostate tissue and in benign prostate hyperplasia [3,4]. Several authors have correlated the expression of the receptor with Gleason score, i.e., high GRPR expression at low Gleason scores and low expression at high Gleason scores [5,20,21,22]. Accordingly, the application of anti-GRPR radioligands may represent a valid complementary approach to prostate-specific membrane antigen (PSMA) targeting for diagnosis and treatment of prostate cancer [23]. As mentioned above, the first introduced BBN-like radioligands were based on native motifs, and hence possessed agonistic properties at the GRPR. This implies that they internalized into target cells after receptor binding. This feature was previously considered to be an important prerequisite for high and prolonged tumor uptake [15,18]. During this initial period, several SPECT and PET GRPR-directed agents were designed for tumor diagnosis with a strong focus on the choice of linker and chelator to optimize pharmacokinetics, and a few of them were tested in patients (Table 1). It should be noted that information on peptide doses used in these studies, along with dosimetry data (where available) are not included or discussed herein, and the reader is advised to seek more detailed information in the references provided. The same holds true for Table 2 (vide infra). 

One of the first SPECT tracers developed for such purposes was [^99m^Tc]Tc-RP527 (RP527, N_3_S-Gly-5-Ava-BBN(7–14)), whereby, the N_3_S donor atom set, attached at the N-terminus via a Gly-5-aminovaleric acid linker, was used for binding Tc-99m under formation of a stable, neutral [^99m^Tc]Tc^V^O(N_3_S) complex in square pyramidal configuration [38]. The tracer was assessed in a small cohort of prostate and breast cancer patients and successfully visualized four out of six breast cancers and one out of four androgen-resistant bone-metastasized prostate carcinomas [24]. Hence, the feasibility of GRPR-targeted imaging of human tumors was established for the first time, despite the shortcomings of the tracer with regards to pharmacokinetics.

Several more GRPR-specific tracers have been developed for SPECT imaging, but very few have been evaluated in the clinic. Suboptimal tumor uptake, in part as a result of poor metabolic stability, and excessive hepatobiliary excretion, due to high lipophilicity, have been the most frequent problems of these compounds [15]. Excretion of [^99m^Tc]Tc-based radiotracers rapidly through the kidneys into urine to minimize radioactivity levels in the abdomen could be promoted by using hydrophilic metal chelates. For such purposes, open chain tetraamines were soon coupled at the N-terminus of bombesin analogs directly or via different linkers. Acyclic tetraamines wrap around the *trans*-[^99m^Tc]Tc^V^(O)_2_^+^ core at the equatorial plane of an octahedron forming monocationic hydrophilic *trans*-[[^99m^Tc]Tc^V^(O)_2_(N_4_)]^+^ complexes [39]. Following this approach, 1,4,8,11-tetraazaundecane via a carboxy anchor at position 6 was covalently coupled to Pro^1^ of full-length [Pro^1^,Nle^14^-NH_2_]BBN affording DB4. The forming hydrophilic radioligand [^99m^Tc]Tc-DB4 showed swift and high localization in human prostate cancer PC-3 xenografts in mice combined with a rapid background clearance predominantly via the kidneys [40]. In a subsequent phase 1 clinical trial in prostate cancer patients, [^99m^Tc]Tc-DB4 successfully imaged primary prostate cancer on SPECT/CT in therapy-naive patients, but had limited efficacy in visualizing pathological lesions in more advanced hormone refractory prostate cancer patients [25]. 

In the PRRT arena, the potent bombesin agonist AMBA (DOTA-Gly-4-aminobenzoyl-BBN(7–14)) has attracted much attention, whereby, the universal chelator DOTA (1,4,7,10-tetraazacyclododecane-1,4,7,10-tetraacetic acid) was coupled to Gln^7^ of BBN(7–14) via a Gly-4-aminobenzoyl linker, thereby, allowing for stable binding of clinically relevant radiometals, including ß ^-^ and α emitters for radionuclide therapy [41]. Accordingly, [^177^Lu]Lu-AMBA was first reported to show high therapeutic potential in several prostate cancer models having different GRPR expression levels, namely based on PC-3, LnCaP, and DU145 cells [42]. Furthermore, the therapeutic efficacy of [^213^Bi]Bi-AMBA was studied and compared with another bombesin radioligand differing only in the linker and labeled both with Lu-177 and Bi-213, [^177^Lu]Lu/[^213^Bi]Bi-DOTA-PESIN (DOTA-PESIN, DOTA-PEG_4_-BBN(7–14)), in PC-3 xenografts [43]. The study reported a superior treatment outcome of the α^−^-therapy as compared with the α-therapy and, among the two [^213^Bi]Bi-radioligands, a better safety profile for [^213^Bi]Bi-DOTA-PESIN than for [^213^Bi]Bi-AMBA. 

Nevertheless, AMBA continues to be the bombesin analog most extensively tested in registered clinical trials, either radiolabeled with Ga-68 as a PET tracer, or radiolabeled with Lu-177 as radiotherapeutic treatment. Interestingly, in a small cohort of patients with different cancer types, [^68^Ga]Ga-AMBA was reported to be tolerated with minor adverse effects (abdominal discomfort and tachycardia), revealing several pathological lesions, especially in breast and prostate cancer patients, and displaying significant uptake mainly in the pancreas and intestinal tract as well as in the esophago-gastric junction [26]. However, a phase I escalation study conducted in patients with metastatic castration-resistant prostate cancer was discontinued due to the severe adverse effects induced by GRPR activation after injection of therapeutic doses of [^177^Lu]Lu-AMBA [27]. 

In conclusion, the application of radiolabeled BBN-like agonists in human, especially during PRRT requiring higher peptide doses administered to patients, has been linked to biosafety hurdles. Although receptor activation following agonist binding triggers internalization of the receptor-(radio)peptide complex into target cells, at the same time severe side effects can be released, predominantly in the gastrointestinal system [14,44,45]. Furthermore, bombesin and its analogs have been reported for their mitogenic actions [46,47]. Consequently, another paradigm was urgently needed for theranostic management of GRPR-positive cancers with this issue being promptly addressed by the advent of radiolabeled GRPR antagonists (vide infra).

## 3. From Agonists to Antagonists in GRPR-Targeting Radioligand Development

**Figure 2:**
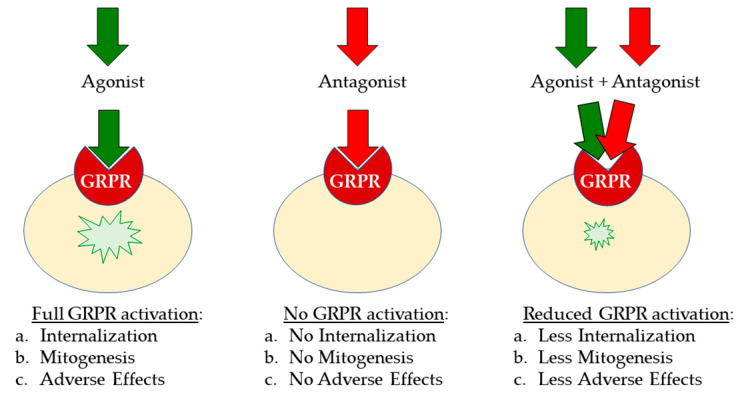
After binding, agonists activate the GRPR on the target-cell membrane, whereas antagonists do not. Antagonists compete with agonists for GRPR binding, and thereby (partially) ban the agonist-induced activation of the GRPR.

Several GRPR antagonists have been developed in previous years in order to better understand the pharmacology and roles of bombesin and its receptors in various physiological and pathophysiological conditions. The growth stimulatory effects of bombesin agonists in several human cancers have triggered systematic efforts to develop GRPR antagonists as antitumoral drugs, capable of binding to the receptor with a high affinity without it being activated [48,49,50]. As summarized in Figure 2, antagonists are not expected to elicit any adverse effects after injection in humans, possessing better biosafety [20,51].

**Figure 3:**
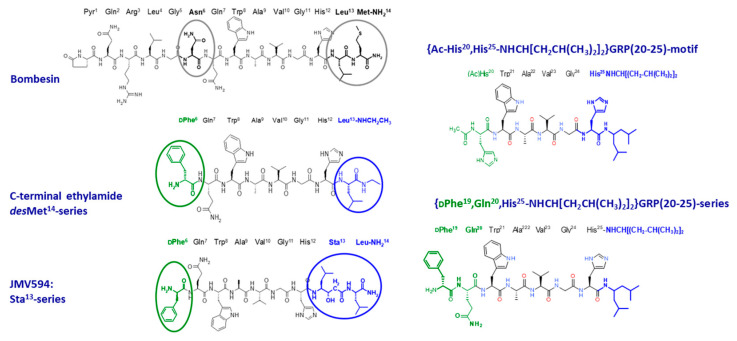
Major BBN- and GRP-based motifs used in the GRPR antagonist design.

Extensive structure-activity relationship studies have resulted in different classes of GRPR-antagonists, generated by modifying the peptide backbone of native BBN or GRP motifs, and especially the C-terminal Leu-Met-NH_2_ dipeptide [1]. The most prominent modifications comprised truncation of the last C-terminal Met and alkyl-amidation or esterification of the exposed carboxy group of the penultimate residue, reduction of peptide bonds, substitution of key amino acids by their D-counterparts or other residues, all leading to a plethora of potent GRPR antagonists, displaying antiproliferative activity in GRPR-expressing cells as well as in GRPR-expressing tumors in mice [52,53]. This rich library of well-characterized compounds has provided a multitude of motifs for the development of radiolabeled GRPR antagonists (GRPR radioantagonists) for application in nuclear medicine. Again, in addition to selecting the most promising peptide motif, other modifications become crucial, such as the choice of the appropriate chelator and radiometal for the intended application and the use or not of a suitable linker, for making available new radioligands with high potential for clinical translation [51]. Representative families of GRPR antagonist-based radioligands, classified according to the peptide motif with clinical interest are discussed below, with relevant structures shown in Figure 3. A list of GRPR antagonist-based radioligands tested in patients is included in Table 2.

### 3.1. Radioligands Based on the GRPR Antagonist [DPhe^6^,Leu^13^-NHEt]BBN(6–13) Motif

Despite the increasing use of PET tracers in clinical practice, medical diagnostic imaging techniques using [^99m^Tc]Tc-based radiopharmaceuticals still account for approximately 80% of all nuclear medicine procedures. This radionuclide is widely available in pharmaceutical grade in many hospitals via commercial [^98^Mo]Mo/[^99m^Tc]Tc-generators, in low-cost and high specific activity and has ideal physical properties with a half-life of 6 h, an optimal energy of 140 keV for imaging with currently used instrumentation combined with low radiation exposure of patients and personnel [39]. Therefore, it is not surprising that the first attempts were devoted to obtaining GRPR antagonists, radiolabeled with Tc-99m and suitable for SPECT imaging. The potent GRPR antagonist [DPhe^6^,Leu^13^-NHEt]BBN(6–13), obtained by ethylamidation of C-terminal Leu^13^ after removal of the last amino acid ([*des*-Met^14^]-class of GRPR antagonists) [54] was conjugated to an acyclic tetraamine chelator via the *p*-aminomethylaniline-diglycolic acid (AMA-DGA) linker. The forming radioligand [^99m^Tc]Tc-Demobesin 1 ([^99m^Tc]Tc-DB1) was the first radiolabeled GRPR antagonist tested in PC-3 tumor-bearing mice showing excellent and specific receptor targeting properties, combining high accumulation in the experimental tumor with a fast background clearance via the kidneys into urine. Rapid washout was observed even from GRPR-rich organs, such as the pancreas, resulting in improved tumor-to-background ratios over time, a feature characteristic for most GRPR radioantagonists thereafter [55]. In a following side-by-side comparison of [^99m^Tc]Tc-DB1 with the agonist-based [^99m^Tc]Tc-DB4, [^99m^Tc]Tc-DB1, despite the lack of internalization, achieved higher tumor uptake and faster washout from GRPR-positive tissues [56]. It should be noted that efforts to prolong tumor retention involved the introduction of different linkers and amino acid replacements, eventually revealing the Sar^11^-analog [^99m^Tc]Tc-DB15 as a promising candidate for clinical translation [28,57]. The first results in patients with advanced breast cancer revealed an excellent pharmacokinetic profile of [^99m^Tc]Tc-DB15, whereby, the tracer visualized several bone and soft tissue metastases on SPECT/CT, while being well tolerated [28]. 

With the aim to obtain a theranostic tool labeled with either diagnostic or therapeutic radiometals, the above GRPR antagonist was coupled to the universal chelator DOTA, affording SB3 (SB3, DOTA-AMA-DGA-[DPhe^6^,Leu^13^-NHEt]BBN(6–13), Table 2). During preclinical testing, in tumor-bearing mice, [^67^Ga]Ga-SB3, serving as a surrogate of [^68^Ga]Ga-SB3, compared well with [^99m^Tc]Tc-DB1, with regards to tumor uptake and retention and background clearance. [^68^Ga]Ga-SB3 was selected for clinical testing in a small cohort of patients with disseminated prostate and breast cancers, whereby it was able to visualize lesions in about 50% of the patients on PET/CT [29]. A much higher detection accuracy was subsequently documented in therapy-naïve primary prostate cancer patients. Furthermore, an excellent correlation could be established between imaging findings and GRPR expression levels by histopathology of excised lesions [21]. Unfortunately, both the SPECT [^111^In]In-SB3 counterpart and the therapeutic [^177^Lu]Lu-SB3 version were shown to be rapidly catabolized in vivo by neutral endopeptidase. This handicap, compromising their clinical translation perspectives, could be, however, overcome by means of specific inhibitors (vide infra) [58]. Indeed, [^111^In]In-SB3 administered in combination with such an inhibitor in mice, successfully visualized PC-3 tumors using SPECT/MRI, revealing its potential for preoperative imaging of prostate cancer [59].

### 3.2. Radioligands Based on the GRPR Antagonist [DPhe^6^,Sta^13^,Leu^14^NH_2_]BBN(6–14) Motif

Replacement of the C-terminal Leu^13^-Met^14^-NH_2_ by the dipeptide Sta^13^-Leu^14^-NH_2_ (Sta or statine, [3S, 4S]-4-amino-3-hydroxy-6-methylheptanoic acid) in the truncated BBN(7–14) motif, led to the potent “statine-based” GRPR antagonist JMV594 [60]. A variety of chelators and linkers have been introduced at its N-terminal to modulate the receptor binding affinities, metabolic stability, hydrophilicity, and hence the end-pharmacokinetics of resulting GRPR radioantagonists. Initially, [^111^In]In-RM1 (RM1, DOTA-Gly-4-aminobenzoyl-JMV594) was developed with DOTA coupled to JMV594 via the same linker used in [^111^In]In-AMBA to allow for a side-by-side comparison [61]. The antagonist showed higher accumulation in PC-3 xenografts and faster washout from the GRPR-positive organs in mice as compared with the agonist, confirming the superior performance of the GRPR antagonist. Intensive structure-activity studies have led to the development of a considerable number of radiolabeled JMV594 derivatives. 

A positive charge at the N-terminus, introduced either by a positively charged linker or by the radiometal chelate per se, turned out to favor GRPR affinity as well as in vivo pharmacokinetics of resulting radioligands [62,63,64,65]. Thus, by using a positively charged 4-amino-1-carboxymethyl-piperidine (Pip) linker, instead, RM2 (DOTA-Pip-[DPhe^6^,Sta^13^,Leu^14^-NH_2_]BBN(6–14), Table 2) was suitable for labeling with Ga-68. [^68^Ga]Ga-RM2 has been widely studied in different prostate cancer cell lines and tumor models and has shown improved binding affinity to the GRPR and metabolic stability [63]. [^68^Ga]Ga-RM2 was the most clinically tested PET tracer based on this class of GRPR antagonists, studied in a small cohort of healthy volunteers [66], breast [30], and prostate cancer patients [31]. In these two latter studies, [^68^Ga]Ga-RM2 was well tolerated by all patients and showed high sensitivity, specificity, and accuracy for the detection of primary cancer. Its feasibility for detecting most metastatic lesions highlights that it has high potential as a diagnostic PET tracer for GRPR-positive tumors. In another approach, NOTA (1,4,7-triazacyclononane-1,4,7-triacetic acid) was attached at the N-terminus of JMV594 either directly or via a PEG_x_ linker, allowing labeling with Ga-68 and the formation of the PET tracer [^68^Ga]Ga-RM26 (RM26, NOTA-PEG_3_-[DPhe^6^,Sta^13^,Leu^14^-NH_2_]BBN(6–14), Table 2) [67]. The tracer successfully (85%) visualized pathological lesions in breast cancer patients, showing significantly more intensive uptake in estrogen receptor (ER)+ patients. Furthermore, an excellent correlation could be established between lesion uptake and GRPR expression status [32]. In patients with primary prostate cancer, the tracer detected prostate confined pathological lesions with an excellent correlation with GRPR expression status in the excised specimens. The tracer was also able to visualize several bone and lymph node metastases in patients with recurring disease [33]. 

Recently, the therapeutic counterpart [^177^Lu]Lu-RM2 has been studied in patients with metastatic castration-resistant prostate cancer, showing promising features for clinical use. Thus, absorbed doses in tumor lesions were found to be therapeutically relevant, whereas rapid clearance from normal GRPR-rich organs was confirmed, such as the pancreas, considered to be the dose-limiting organ due to its high radioligand uptake [37]. In addition, positively charged radiometal chelates, formed after stable binding of specific radiometals of clinical interest to chelators other than DOTA, were also considered [62,68]. Thus, in an interesting study, the GRPR antagonist AR (H-PEG_4_-JMV594) carrying four different radiometal chelates on its N-terminus ([^111^In]In-DOTA, [^99m^Tc]Tc-N_4_, [^68^Ga]Ga-NODAGA, and [^64^Cu]Cu-CB-TE2A), confirmed the superiority of positively charged conjugates ([^99m^Tc]Tc-N_4_-AR and [^64^Cu]Cu-CB-TE2A-AR) in terms of GRPR affinity and tumor uptake and retention. Notably, [^64^Cu]Cu-CB-TE2A-AR (CB-TE2A, 4,11-bis(carboxymethyl)-1,4,8,11-tetraazabicyclo(6.6.2)hexadecane, Table 2) was finally chosen for further clinical evaluation also owing to the attractive nuclear properties of Cu-64. Indeed, the half-life of 12.7 h and its decay scheme render this theranostic radiometal an attractive alternative to the usually applied PET radionuclides F-18 and Ga-68 [69]. In a first proof-of-concept study in a small number of prostate cancer patients [^64^Cu]Cu-CB-TE2A-AR showed long tumor retention and fast clearance from other organs [36].

### 3.3. Radioligands Based on the GRPR Antagonist [Ac-His^20^,His^25^-NHR]GRP(20–25) Motif

**Figure 4:**
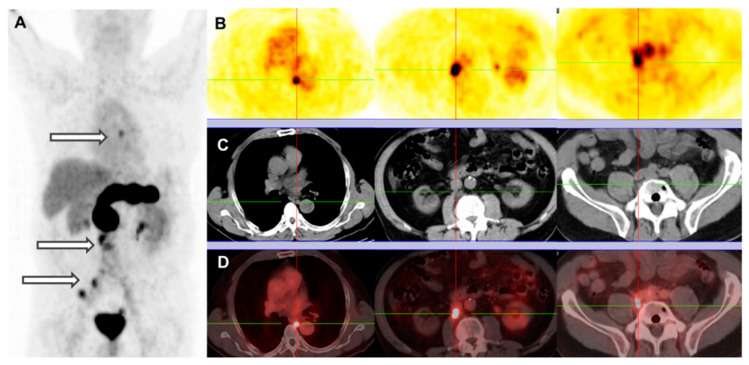
Patient with PC, post radical prostatovesiculectomy with pelvic lymphadenectomy, IMRT, and androgen deprivation therapy (PSA on date of scan, 21.77 ng/mL). [^68^Ga]Ga-NeoBOMB1 PET/CT: (**A**) PET MIP; (**B**) serial PET transverse; (**C**) corresponding CT transverse; (**D**) fusion PET/CT images. Multiple mediastinal, abdominal, paraoesophageal and pelvic lymph node metastatses (arrows and crossbars). This research was originally published in *JNM* 2017 [34].

Alkylamide derivatives of the human [(N-acetyl)His^20^,His^25^-NHR,*des*-(Leu^26^,Met^27^NH_2_)]GRP(20–27) motif (especially when NHR = NHCH(CH_2_CH(CH_3_)_2_)_2_) represent a successful class of very potent GRPR antagonists, characterized by high receptor affinity and in vivo enzymatic stability. Accordingly, the [DPhe^19^,Gln^20^,His^25^-NHCH(CH_2_CH(CH_3_)_2_)_2_]GRP(20–25) sequence was modified by DOTA-AMA-DGA at the N-terminus affording NeoBOMB1. NeoBOMB1, labeled with Ga-68 (for PET), In-111 (for SPECT), or Lu-177 (for PRRT) showed promising theranostic profiles during preclinical evaluation, characterized by high receptor affinity and cell-uptake efficiency in vitro together with high metabolic stability and high and prolonged tumor uptake in GRPR-expressing tumors in vivo [34,70,71]. First clinical data with [^68^Ga]Ga-NeoBOMB1 in a group of prostate cancer patients highlighted its ability to visualize primary tumors as well as liver metastases and bone lesions (example in Figure 4). These promising outcomes justified the assessment of the theranostic pair [^68^Ga]Ga/[^177^Lu]Lu-NeoBOMB1 in an ongoing multicenter clinical trial (NCT03872778) in patients with advanced solid tumors known to overexpress GRPR [35].

## 4. Metabolic Stability Issues

A major handicap in the development of peptide-based radioligands for cancer theranostics is the notorious susceptibility of peptides to proteolytic degradation [72,73,74,75]. Omnipresent peptidases, i.e., enzymes breaking down peptide bonds, can drastically reduce the number of intact radiopeptide molecules eventually reaching the tumor-situated target. As a result, tumor uptake drops and, consequently, diagnostic accuracy and therapeutic efficacy are compromised [76]. It should be noted that amongst the >550 proteases comprising the human protease degradome [77], the circulating radiopeptide will encounter quite a few to which it may potentially be a substrate on its quick trip to the target. Another point to keep in mind is that most peptide radioligands carry the radiometal chelate at the N-terminus (either directly or via a spacer), and therefore are resistant to the proteolytic action of ubiquitous N-aminopeptidases.

**Figure 5:**
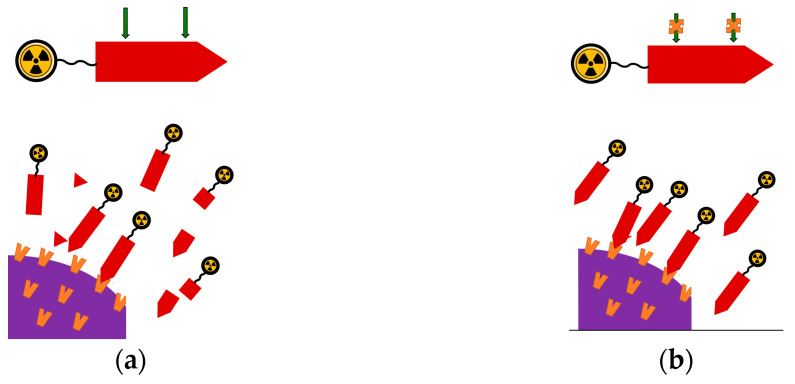
(**a**) The radiolabeled peptide conjugate (based on a GRPR agonist or antagonist) after entering the blood (top) is attacked by NEP (indicated by the two descending green arrows) and is degraded. As a result, only a few of the initial molecules arrive intact at the tumor cells and interact with the GRPR (orange receptors) located at the cell membrane (bottom); (**b**) following co-administration of a NEP inhibitor, the degrading action of NEP is suspended, and thus the number of intact molecules arriving at the tumor and binding to GRPR sites is higher, leading to higher tumor uptake.

The performance of BBN-like radioligands is likewise affected by rapidly degrading peptidases in the blood stream (Figure 5a). Previous studies have revealed neprilysin (NEP) as the major player in the degradation of radiolabeled BBN-like analogs [76,78,79]. NEP is an ectoenzyme found in high local concentrations anchored on the membrane of epithelial cells of the vasculature and major organs of the body, such as the kidneys, the lungs, and the intestinal track. Typically, NEP hydrolyzes peptide bonds at the amino side of hydrophobic residues and shows a wide substrate repertoire, including BBN-like peptides [80,81]. Four cleavage sites have been, thus far, identified in the BBN(6/7–14) motif most often present in anti-GRPR radioligands, namely the His^12^-Leu^13^, Ala^9^-Val^10^, Trp^8^-Ala^9^, and Gln^7^-Trp^8^ bonds [58,79].

Several structural strategies toward metabolically robust anti-GRPR radiopeptides have been pursued over the years. Substitution of selected amino acids by unnatural residues, such as Gly^11^ (by DAla^11^, Sar^11^ (Sar, sarcosine), N-methylglycine, or beta-Ala^11^) and/or Gln^7^ (by DGln^7^) being the most common, was successful in improving metabolic stability of resulting radioligands at the cost, however, of other important pharmacological properties, such as cell uptake, BBN-receptor subtype selectivity, and pharmacokinetics [57,58,76,82]. The same holds true for other structural interventions, such as the use of multimeric peptide versions [83,84,85]. In another recent approach, one or more peptide bonds have been substituted by their triazolyl isosteres to improve the metabolic stability of resulting GRPR-directed radiopeptides, but again tumor uptake in animal models could not be significantly improved as compared with the non-modified analogs [86,87,88]. 

In yet another promising approach, NEP inhibitor administration was proposed to ban the action of the major degrading protease (Figure 5b). The resulting in situ stabilization of circulating BBN-like radioligands maximized their delivery to tumor-associated GRPR sites, thereby improving tumor uptake. This strategy turned out to be particularly effective in enhancing the GRPR-targeting capabilities of biodegradable analogs without interfering with other important biological properties, such as cell uptake or pharmacokinetics, but unsurprisingly, was less evident in metabolically more robust analogs [58,76,82,88,89,90,91,92]. Although GRPR radioantagonists turned out to be typically more stable than their agonist-based counterparts, notable tumor uptake improvements could still be observed in most cases, providing new exciting prospects for better diagnosis and therapy [59,93]. In most early preclinical studies testing this approach, phosphoramidon (PA) was used as an NEP inhibitor [76,94,95]. Translation of this methodology in the clinic needs to overcome a series of safety and regulatory hurdles and may be facilitated by the availability of registered and orally administered NEP inhibitors used as anti-diarrhea (Hidrasec^TM^, in vivo releasing the potent and selective NEP-inhibitor thiorphan) or as anti-hypertensive drugs (Entresto^®^, containing the prodrug sacubitril (AHU377), in vivo releasing the active substance sacubitrilat (LBQ657), a potent NEP inhibitor) [96,97,98,99,100,101].

## 5. Newer Trends in the Application of Anti-GRPR Theranostic Radiopeptides

In view of the above, it is fair to conclude that molecular imaging and PRRT of human GRPR-positive tumors has been gaining momentum in radiopharmacy and nuclear medicine research with a few tracers currently being evaluated by multicenter clinical trials for entering the clinical arena. Recent breakthroughs include, on the one hand, the advent of GRPR radioantagonists competently addressing biosafety concerns and, on the other hand, the application of clinically established protease inhibitors or the “smart” design of protease-resistant analogs to improve metabolic stability. Nevertheless, a few critical questions still need to be tackled before drastically improving the diagnostic accuracy and, most importantly, the therapeutic efficacy of this approach. Hence, it is essential to acquire a better understanding of the pathophysiology of GRPR-positive cancers and, especially, the dependence of GRPR expression density on disease type, stage, coexpression of other biomolecular targets, individual patient biochemical background, and previous or even concomitant therapies. 

For example, an alternative biomolecular target in prostate cancer is PSMA, prompting the development of several radiolabeled PSMA inhibitors dynamically entering the field of nuclear medicine as novel diagnostic and therapeutic tools [102]. Heterogeneity of GRPR and/or PSMA expression in primary and metastatic cancer lesions through the various stages of the disease has been documented [9,22,51,103,104]. This finding has triggered the development of heterodimeric radioligands, which are able to target both the GRPR and PSMA in prostate cancer lesions, thereby, increasing diagnostic sensitivity and therapeutic efficacy [84,105,106,107]. Likewise, in another approach, the GRPR and integrin α_V_β_3_ associated with tumor angiogenesis have been concomitantly targeted by a dual PET tracer [^68^Ga]Ga-BBN-RGD enhancing diagnostic accuracy in breast and prostate cancer patients [108,109].

**Figure 6:**
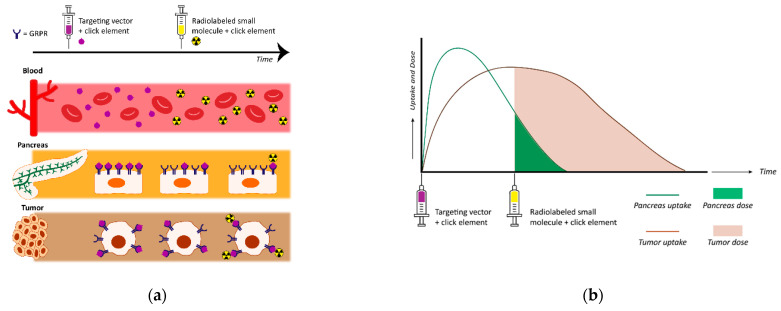
The pretargeting concept for safe and effective GRPR-mediated radionuclide therapy: (**a**) The targeting vector adapted with a click moiety is injected first. It binds to the GRPR on the cells of the pancreas and on GRPR-expressing tumor cells. When the targeting vector is (mostly) cleared from the pancreas but still present on the tumor cells, a radiolabeled small molecule containing a complementary click element is injected. This radiolabeled small molecule couples the targeting vector still present on the tumor cells, hereby, specifically delivering radioactivity to these cells; (**b**) graph depicting uptake of the targeting vector and radioactivity/radiation dose to the pancreas and tumor using the pretargeting strategy.

How the above strategies applied in PET imaging can be adopted in PRRT remains to be explored, given that the issue of inadvertent accumulation of radioactivity in excretory organs or organs with high physiological GRPR expression levels (e.g., the pancreas) have to be competently addressed first. This issue becomes even more relevant in the case of PRRT with alpha emitters [43]. Higher tumor-to-background ratios have been achieved, in preclinical studies, with the application of higher peptide doses, an approach potentially applied in humans only for GRPR antagonists owing to biosafety concerns [70]. Interestingly, an extra benefit of administering higher peptide amounts was recently shown to favor penetration of radioactivity into the whole tumor mass in mice, thereby, improving therapeutic efficacy [110]. In another interesting approach depicted in Figure 6, a pretargeting strategy has been proposed to circumvent pancreatic uptake. Pretargeting strategies have been applied with success to lower tumor-to-background radioactivity ratios of long-circulating high molecular weight molecules, for example, antibodies [111,112]. Preliminary studies in mice with derivatives of NeoBOMB1, carrying a clickable residue, have been applied in combination with the radiolabeled complementary small molecule [^111^In]In-Tz, and thus far, have failed to improve tumor-to-pancreas ratios, most likely due to undesired folding of the targeting vector in the binding pocket of the GRPR [113]. Therefore, targeting vectors with longer linkers are currently being developed and preclinically tested, aiming to prevent pancreatic radiotoxicity, and thus enabling higher radioactivity doses to the tumor.

Several other efforts have been directed to enhance the therapeutic efficacy of PRRT in GRPR-positive tumors via adjuvant therapeutic schemes. Thus, the efficacy of monotherapy with [^177^Lu]Lu-RM2 alone in mice models has been reported to further improve when combined with the mTOR inhibitor rapamycin [114]. Interestingly, mTOR inhibitors were reported to enhance the expression levels of other GPCRs in experimental tumors, revealing the need for systematic studies in this promising area of research [115]. The combination of PRRT and hormone therapy in breast and prostate cancer may provide another promising route toward higher therapeutic responses [116]. Immunotherapy may also enhance therapeutic efficacy of PRRT, as recently shown for the [^177^Lu]Lu-RM26 and trastuzumab combination in prostate cancer mice models [110]. In a recent innovative approach, prolonged tumor retention could be achieved in mice models when using GRPR peptide radioligands, either agonists or antagonists, modified with cysteine cathepsin inhibitors, via an endolysosomal trapping mechanism [117].

Last but not least, one should report recent breakthroughs in the production of clinically appealing therapeutic radionuclides, including the Tb and Sc therapeutic isotopes and alpha emitters (Ac-225 and Bi-213), dynamically entering radiopharmaceutical development, and expected to soon upgrade the arsenal of anti-GRPR therapeutics with new powerful molecular tools [118,119,120].

## 6. The Clinician’s View: GRPR-Targeted Theranostics with a Focus on Prostate Cancer

In the last decade, new developments in imaging of prostate cancer have had a major impact on the daily practice of prostate cancer specialists. To put things in perspective, it is important to realize that guidelines are still heavily based on results of two large prostate cancer screening trials (ERSPC and PLCO), starting in the late 1980s with only PSA, rectal examination, low-resolution ultrasound, and six random biopsies as screening tools [121]. Two big game changers can be depicted that have significantly reformed the management of prostate cancer, i.e., MRI and (PSMA-) PET (or PET/CT), improving the average prognosis [122,123]. However, PSMA is not as specific as we wish and several other malignant, benign, and non-clinically relevant conditions might be mistaken for a prostate cancer lesion and can lead to false conclusions [124,125]. Furthermore, limitations of PSMA are also apparent with respect to the relative low sensitivity for early stage, low-grade tumors, or early recurrent disease, thereby impairing decision making in patients with oligo-metastatic disease, whereby the exact number and localization of metastatic lesions is of great importance [126]. Accordingly, PET imaging of other molecular targets, such as GRPR, may well contribute to this clinical need of refining decision making [104].

**Figure 7:**
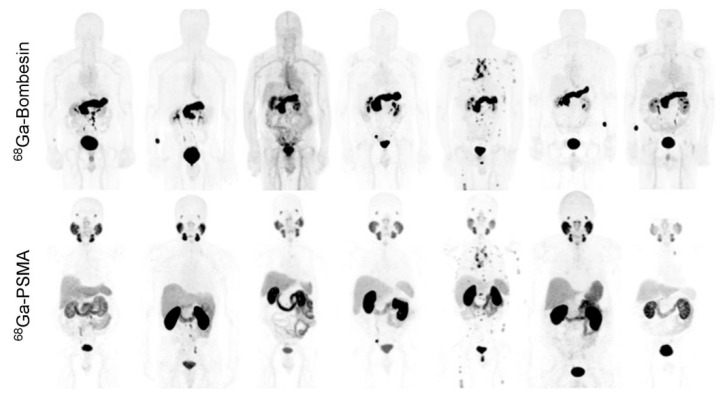
Maximum intensity projection [^68^Ga]Ga-RM2 and [^68^Ga]Ga-PSMA-11 images of the 7 enrolled prostate cancer patients. This research was originally published in *JNM* 2016 [103].

GRPR-targeted imaging may constitute a relevant addition for those patients with PSMA-negative tumors, estimated to constitute 74% of patients, or those with low-grade tumors that do not show on MRI nor PSMA PET scans [127]. Indeed, retrospective studies have revealed that PSMA expression was inversely correlated with GRPR, underscoring the potential value of their combined use [22]. Recent phase I studies have confirmed GRPR PET imaging to detect (primary) prostate tumors with high sensitivity and specificity comparable to those observed for PSMA [59]. Further reports have shown that PSMA and GRPR PET/CT may have added value in evaluating biochemical recurrence of prostate cancer [103,104]. In the direct comparison of [^68^Ga]Ga-RM2 and [^68^Ga]Ga-PSMA-11 on PET/CT in seven patients with biochemically recurring prostate cancer, shown in Figure 7, the heterogeneity of GRPR and PSMA expression was evident, highlighting the benefit of using both targets in a complementary fashion to increase diagnostic accuracy [103]. Promising new developments are directed towards bispecific radioligands of PSMA and GRPR to reach maximal detection rates and to capture all prostate cancer lesions [84,105,106,107]. 

Importantly, GRPR and PSMA expression are both affected by standard of care treatments applied in prostate cancer, including targeting the androgen receptor pathway and taxane-based chemotherapy [29]. Although reported data are contradictory on the effect size, this clearly constitutes a serious issue for clinicians. As a consequence of the multitude of options for treatment of disseminated prostate cancer [128], late-stage patients have highly variable treatment histories that jeopardize good clinical evaluation of the most optimal timing of GRPR and PSMA imaging (and therapy) in these patients.

In addition to their use in diagnostics, PSMA and GRPR hold promise for the application of radionuclide therapy in the management of prostate cancer. Clinical application of this theranostic approach has just started, and reports are still scarce [129]. Although several spectacular cases have been presented with seemingly complete remissions after treatment with PSMA or GRPR, as well as promising data achieved in several retrospective studies, robust scientific proof of efficacy and long-term safety in randomized clinical trials are yet to be completed [37,130,131]. A meta-analysis of 12 studies in late-stage/end-stage patients failing on standard of care treatments, revealed transient effectivity of PSMA radiotherapy, although overall survival benefit was not significantly improved as compared with any other third-line treatment [132,133,134]. From these studies, short-term side effects appear to be minimal for PSMA, but administration of these treatments will not be approved in an early metastasized stage until long term safety is scientifically proven. Likewise, GRPR expression is also not unique to the prostate nor to prostate cancer; the high absorbed pancreatic dose and diffuse low uptake throughout the gastrointestinal tract constitute a serious concern for therapy [21,135]. With new developments in alpha radionuclide therapy, toxicity in surrounding tissues will become even more critical. Despite major consequences of PSMA radiotherapy with loss of saliva, taste, speech, and dry eyes, [^225^Ac]Ac-PSMA therapy is already being evaluated in the clinic with promising biochemical responses and low treatment-related toxicity [136]. Finally, tandem therapy with both [^177^Lu]Lu- and [^225^Ac]Ac-PSMA have been applied that suggest improving PSMA-radiotherapy response while minimizing toxicity of the salivary glands [133]. Alternatively, combination strategies of radiotherapy with both PSMA and GRPR are being considered as a solution to mitigate toxicity while optimizing antitumor efficacy [137].

The fast introduction of PSMA radiotracers for imaging and therapy has radically shifted the clinical perspectives of prostate cancer patients. But clearly, clinical evaluation has just started, and well-designed prospective randomized trials are needed to learn how to use these new applications in daily practice: (1) how to interpret PSMA PET/CT (e.g., the clinical relevance of lesions, how to deal with PSMA-negative tumors); (2) how to optimally position PSMA radiotherapy in the sequence of standard of care therapies; and most importantly, (3) how to alleviate radiotoxicity without losing therapeutic efficacy. GRPR-targeted radioligands may help to solve, at least in part, these clinical issues by complementing the PSMA PET/CT and radioligand therapy and improving the prostate cancer detection rate as well as therapeutic efficacy.

## 7. Conclusions

In view of the above, it becomes evident that research in the field of radiolabeled bombesin analogs for application in the theranostic management of GRPR-positive human tumors has been thriving with major breakthroughs already achieved. Firstly, the paradigm shift from agonist- to antagonist-based radioligands has competently addressed the issue of biosafety, a very crucial point during PRRT with higher peptide amounts administered to patients. Secondly, the challenge of rapid degradation of BBN-like tracers entering the circulation has been “elegantly” met with the application of NEP inhibitors. The latter may be directly applied for in situ stabilization of biodegradable radioligands, but at the same time have been instrumental in a smarter design of metabolically robust analogs. Extended and systematic structure-activity relationship studies have pinpointed candidates eligible for clinical translation in breast, prostate, and other GRPR-positive cancer patients, applying SPECT/CT, or PET/CT imaging and PRRT.

Such pioneer clinical studies have been eye-openers with regards to the strengths and limitations of GRPR-positive cancer theranostics, revealing new challenges to be met. Our incomplete understanding of cancer pathology needs to be improved in order to provide solid information on critical issues, such as GRPR expression status in different types and stages of cancer, as well as following previous therapies in cases of advanced disease. This critical knowledge will certainly provide new opportunities for the successful application of multitargeted theranostic agents and, most importantly, of adjuvant therapies. Accordingly, combining PRRT with the aid of anti-GRPR radioligands with other therapies, such as hormone therapy, immunotherapy, and other therapeutic schemes, should be adopted to individual patients, aiming at maximum efficacy and low toxicity. The seeds for these innovative developments have already been planted and will most certainly lead to new exciting breakthroughs in the near future. 

## Figures and Tables

**Table 1:** Bombesin-based agonists tested in the clinic. BBN(7–14), H-Gln-Trp-Ala-Val-Gly-His-Leu-Met-NH_2_.

BBN Analog	Radioconjugate Sequence	Clinical Indication	Ref.
**SPECT Tracers**			
[^99m^Tc]Tc-RP527	[^99m^Tc]Tc-N_3_S-Gly-5aVa^†^-BBN(7–14)	Metastasized PC^§^ or BC^€^	[24]
[^99m^Tc]Tc-DB4	[^99m^Tc]Tc-N_4_-[Pro^1^,Nle^14^]BBN	PC	[25]
**PET Tracers**			
[^68^Ga]Ga-AMBA	[^68^Ga]Ga-DOTA-Gly-4-aminobenzyl-BBN(7–14)	Different malignancies	[26]
**PRRT Agents**			
[^177^Lu]Lu-AMBA	[^177^Lu]Lu-DOTA-Gly-4-aminobenzyl-BBN(7–14)	Metastatic castration-resistant PC	[27]

^†^5aVa, 5-aminovaleric acid; ^§^PC, prostate cancer; ^€^BC, breast cancer.

**Table 2:** GRPR-antagonist-based peptide radioligands tested in the clinic. BBN(7–14), H-Gln-Trp-Ala-Val-Gly-His-Leu-Met-NH_2_.

Radioligand	Radioconjugate Sequence	Clinical Indication	Ref.
**SPECT Tracers**			
[^99m^Tc]Tc-DB15	[^99m^Tc]Tc-N_4_-AMA^‡^-DGA^†^-[DPhe^6^,Sar^11^,Leu^13^-NHEt]BBN(6–13)	Advanced BC^⇈^	[28]
**PET Tracers**			
[^68^Ga]Ga-SB3	[^68^Ga]Ga-DOTA-AMA^‡^-DGA^†^-[DPhe^6^,Leu^13^-NHEt]BBN(6–13)	Disseminated PC^⇊^, BC^⇈^	[21,29]
[^68^Ga]Ga-RM2	[^68^Ga]Ga-DOTA-Pip^§^-[DPhe^6^,Sta^13^,Leu^14^-NH_2_]BBN(6–14)	PC^⇊^, BC^⇈^	[30,31]
[^68^Ga]Ga-RM26	[^68^Ga]Ga-NOTA-PEG_3_-[DPhe^6^,Sta^13^,Leu^14^-NH_2_]BBN(6–14)	PC^⇊^, BC^⇈^	[32,33]
[^68^Ga]Ga-NeoBOMB1	[^68^Ga]Ga-DOTA-AMA^‡^-DGA-[DPhe^19^,Gln^20^,His^25^-NHR]GRP(20–25)^€^	Different malignancies	[34,35]
[^64^Cu]Cu-CB-TE2A	[^64^Cu]Cu-CB-TE2A-PEG_4_-[DPhe^6^,Sta^13^,Leu^14^-NH_2_]BBN(6–14)^#^	Newly diagnosed PC^⇊^	[36]
**PRRT Agents**			
[^177^Lu]Lu-RM2	[^177^Lu]Lu-DOTA-Pip^§^-[DPhe^6^,Sta^13^,Leu^14^-NH_2_]BBN(6–14)	castration-resistant PC^⇊^	[37]
[^177^Lu]Lu-NeoBOMB1	[^177^Lu]Lu-DOTA-AMA^‡^-DGA^†^-[DPhe^19^,Gln^20^,His^25^-NHR]GRP(20–25)^€^	Advanced solid tumors	(NCT03872778)

^‡^ AMA, *p*-aminomethylaniline; ^†^DGA, diglycolic acid; ^§^Pip, 4-amino-1-carboxymethyl-piperidine; ^€^R, -CH(CH_2_CH(CH_3_)_2_)_2_; ^⇊^PC, prostate cancer; ^#^CB-TE2A, 4,11-bis(carboxymethyl)-1,4,8,11-tetraazabicyclo(6.6.2)hexadecane; ^⇈^ BC, breast cancer.

## References

[1] Jensen R.T., Battey J.F., Spindel E.R., Benya R.V. (2008). International union of pharmacology. LXVIII. Mammalian bombesin receptors: Nomenclature, distribution, pharmacology, signaling, and functions in normal and disease states. Pharmacol. Rev..

[2] Moreno P., Ramos-Alvarez I., Moody T.W., Jensen R.T. (2016). Bombesin related peptides/receptors and their promising therapeutic roles in cancer imaging, targeting and treatment. Expert. Opin. Ther. Targets.

[3] Markwalder R., Reubi J.C. (1999). Gastrin-releasing peptide receptors in the human prostate: Relation to neoplastic transformation. Cancer Res..

[4] Körner M., Waser B., Rehmann R., Reubi J.C. (2014). Early over-expression of GRP receptors in prostatic carcinogenesis. Prostate.

[5] Beer M., Montani M., Gerhardt J., Wild P.J., Hany T.F., Hermanns T., Muntener M., Kristiansen G. (2012). Profiling gastrin-releasing peptide receptor in prostate tissues: Clinical implications and molecular correlates. Prostate.

[6] Gugger M., Reubi J.C. (1999). Gastrin-releasing peptide receptors in non-neoplastic and neoplastic human breast. Am. J. Pathol..

[7] Reubi C., Gugger M., Waser B. (2002). Co-expressed peptide receptors in breast cancer as a molecular basis for in vivo multireceptor tumour targeting. Eur. J. Nucl. Med. Mol. Imaging.

[8] Morgat C., MacGrogan G., Brouste V., Velasco V., Sevenet N., Bonnefoi H., Fernandez P., Debled M., Hindie E. (2017). Expression of gastrin-releasing peptide receptor in breast cancer and its association with pathologic, biologic, and clinical parameters: A study of 1432 primary tumors. J. Nucl. Med..

[9] Dalm S.U., Martens J.W., Sieuwerts A.M., van Deurzen C.H., Koelewijn S.J., de Blois E., Maina T., Nock B.A., Brunel L., Fehrentz J.A. (2015). In vitro and in vivo application of radiolabeled gastrin-releasing peptide receptor ligands in breast cancer. J. Nucl. Med..

[10] Mattei J., Achcar R.D., Cano C.H., Macedo B.R., Meurer L., Batlle B.S., Groshong S.D., Kulczynski J.M., Roesler R., Dal Lago L. (2014). Gastrin-releasing peptide receptor expression in lung cancer. Arch. Pathol. Lab. Med..

[11] Reubi J.C., Körner M., Waser B., Mazzucchelli L., Guillou L. (2004). High expression of peptide receptors as a novel target in gastrointestinal stromal tumours. Eur. J. Nucl. Med. Mol. Imaging.

[12] Reubi J.C. (2007). Peptide receptor expression in GEP-NET. Virchows Archiv..

[13] Reubi J.C., Wenger S., Schmuckli-Maurer J., Schaer J.C., Gugger M. (2002). Bombesin receptor subtypes in human cancers: Detection with the universal radioligand ^125^I-[dTyr^6^,beta-Ala^11^,Phe^13^,Nle^14^]bombesin (6–14). Clin. Cancer Res..

[14] Bitar K.N., Zhu X.X. (1993). Expression of bombesin-receptor subtypes and their differential regulation of colonic smooth muscle contraction. Gastroenterology.

[15] Maina T., Nock B.A. (2017). From bench to bed: New gastrin-releasing peptide receptor-directed radioligands and their use in prostate cancer. PET Clin..

[16] Li X., Cai H., Wu X., Li L., Wu H., Tian R. (2020). New frontiers in molecular imaging using peptide-based radiopharmaceuticals for prostate cancer. Front. Chem..

[17] Nock B.A., Maina T. (2019). Theranostic approaches in nuclear oncology: From bench to bed. J. Label. Comp. Radiopharm..

[18] Baratto L., Jadvar H., Iagaru A. (2018). Prostate cancer theranostics targeting gastrin-releasing peptide receptors. Mol. Imaging Biol..

[19] Sung H., Ferlay J., Siegel R.L., Laversanne M., Soerjomataram I., Jemal A., Bray F. (2021). Global cancer statistics 2020: GLOBOCAN estimates of incidence and mortality worldwide for 36 cancers in 185 countries. CA Cancer J. Clin..

[20] Mansi R., Fleischmann A., Mäcke H.R., Reubi J.C. (2013). Targeting GRPR in urological cancers—From basic research to clinical application. Nat. Rev. Urol..

[21] Bakker I.L., Fröberg A.C., Busstra M.B., Verzijlbergen J.F., Konijnenberg M., van Leenders G., Schoots I.G., de Blois E., van Weerden W.M., Dalm S.U. (2021). GRPR antagonist ^68^Ga-SB3 PET/CT-imaging of primary prostate cancer in therapy-naive patients. J. Nucl. Med..

[22] Faviana P., Boldrini L., Erba P.A., Di Stefano I., Manassero F., Bartoletti R., Galli L., Gentile C., Bardi M. (2021). Gastrin-releasing peptide receptor in low grade prostate cancer: Can it be a better predictor than prostate-specific membrane antigen?. Front. Oncol..

[23] Iagaru A. (2017). Will GRPR compete with PSMA as a target in prostate cancer?. J. Nucl. Med..

[24] Van de Wiele C., Dumont F., Vanden Broecke R., Oosterlinck W., Cocquyt V., Serreyn R., Peers S., Thornback J., Slegers G., Dierckx R.A. (2000). Technetium-99m RP527, a GRP analogue for visualisation of GRP receptor-expressing malignancies: A feasibility study. Eur J. Nucl. Med..

[25] Mather S.J., Nock B.A., Maina T., Gibson V., Ellison D., Murray I., Sobnack R., Colebrook S., Wan S., Halberrt G. (2014). GRP receptor imaging of prostate cancer using [^99m^Tc]Demobesin 4: A first-in-man study. Mol. Imaging Biol..

[26] Baum R., Prasad V., Mutloka N., Frischknecht M., Maecke H., Reubi J.C. (2007). Molecular imaging of bombesin receptors in various tumors by Ga-68 AMBA PET/CT: First results. J. Nucl. Med..

[27] Bodei L., Ferrari M., Nunn A., Llull J., Cremonesi M., Martano L., Laurora G., Scardino E., Tiberini S., Bufi G. (2007). ^177^Lu-AMBA bombesin analogue in hormone refractory prostate cancer patients: A phase I escalation study with single-cycle administrations. Eur. J. Nucl. Med. Mol. Imaging.

[28] Nock B.A., Kaloudi A., Kanellopoulos P., Janota B., Bromińska B., Iżycki D., Mikołajczak R., Czepczynski R., Maina T. (2021). [^99m^Tc]Tc-DB15 in GRPR-targeted tumor imaging with SPECT: From preclinical evaluation to the first clinical outcomes. Cancers.

[29] Maina T., Bergsma H., Kulkarni H.R., Mueller D., Charalambidis D., Krenning E.P., Nock B.A., de Jong M., Baum R.P. (2016). Preclinical and first clinical experience with the gastrin-releasing peptide receptor-antagonist [^68^Ga]SB3 and PET/CT. Eur. J. Nucl. Med. Mol. Imaging.

[30] Stoykow C., Erbes T., Maecke H.R., Bulla S., Bartholomä M., Mayer S., Drendel V., Bronsert P., Werner M., Gitsch G. (2016). Gastrin-releasing peptide receptor imaging in breast cancer using the receptor antagonist ^68^Ga-RM2 and PET. Theranostics.

[31] Kähkonen E., Jambor I., Kemppainen J., Lehtio K., Gronroos T.J., Kuisma A., Luoto P., Sipila H.J., Tolvanen T., Alanen K. (2013). In vivo imaging of prostate cancer using [^68^Ga]-labeled bombesin analog BAY86-7548. Clin. Cancer Res..

[32] Zang J., Mao F., Wang H., Zhang J., Liu Q., Peng L., Li F., Lang L., Chen X., Zhu Z. (2018). ^68^Ga-NOTA-RM26 PET/CT in the evaluation of breast cancer: A pilot prospective study. Clin. Nucl. Med..

[33] Zhang J., Niu G., Fan X., Lang L., Hou G., Chen L., Wu H., Zhu Z., Li F., Chen X. (2018). PET using a GRPR antagonist ^68^Ga-RM26 in healthy volunteers and prostate cancer patients. J. Nucl. Med..

[34] Nock B.A., Kaloudi A., Lymperis E., Giarika A., Kulkarni H.R., Klette I., Singh A., Krenning E.P., de Jong M., Maina T. (2017). Theranostic perspectives in prostate cancer with the gastrin-releasing peptide receptor antagonist NeoBOMB1: Preclinical and first clinical results. J. Nucl. Med..

[35] Djaileb L., Morgat C., van der Veldt A., Virgolini I., Cortes F., Demange A., Orlandi F., Wegener A. (2020). Preliminary diagnostic performance of [^68^Ga]-NeoBOMB1 in patients with gastrin-releasing peptide receptor-positive breast, prostate, colorectal or lung tumors (Neofind). J. Nucl. Med..

[36] Wieser G., Mansi R., Grosu A.L., Schultze-Seemann W., Dumont-Walter R.A., Meyer P.T., Maecke H.R., Reubi J.C., Weber W.A. (2014). Positron emission tomography (PET) imaging of prostate cancer with a gastrin releasing peptide receptor antagonist—From mice to men. Theranostics.

[37] Kurth J., Krause B.J., Schwarzenbock S.M., Bergner C., Hakenberg O.W., Heuschkel M. (2020). First-in-human dosimetry of—Gastrin-releasing peptide receptor antagonist [^177^Lu]Lu-RM2: A radiopharmaceutical for the treatment of metastatic castration-resistant prostate cancer. Eur. J. Nucl. Med. Mol. Imaging.

[38] Smith C.J., Gali H., Sieckman G.L., Higginbotham C., Volkert W.A., Hoffman T.J. (2003). Radiochemical investigations of ^99m^Tc-N_3_S-X-BBN[7–14]NH_2_: An in vitro/in vivo structure-activity relationship study where X = 0-, 3-, 5-, 8-, and 11-carbon tethering moieties. Bioconjug. Chem..

[39] Nock B., Maina T. (2012). Tetraamine-coupled peptides and resulting ^99m^Tc-radioligands: An effective route for receptor-targeted diagnostic imaging of human tumors. Curr. Top. Med. Chem..

[40] Nock B.A., Nikolopoulou A., Galanis A., Cordopatis P., Waser B., Reubi J.C., Maina T. (2005). Potent bombesin-like peptides for GRP-Receptor targeting of tumors with ^99m^Tc: A preclinical study. J. Med. Chem..

[41] Lantry L.E., Cappelletti E., Maddalena M.E., Fox J.S., Feng W., Chen J., Thomas R., Eaton S.M., Bogdan N.J., Arunachalam T. (2006). ^177^Lu-AMBA: Synthesis and characterization of a selective ^177^Lu-labeled GRP-R agonist for systemic radiotherapy of prostate cancer. J. Nucl. Med..

[42] Maddalena M.E., Fox J., Chen J., Feng W., Cagnolini A., Linder K.E., Tweedle M.F., Nunn A.D., Lantry L.E. (2009). ^177^Lu-AMBA biodistribution, radiotherapeutic efficacy, imaging, and autoradiography in prostate cancer models with low GRP-R expression. J. Nucl. Med..

[43] Wild D., Frischknecht M., Zhang H.W., Morgenstern A., Bruchertseifer F., Boisclair J., Provencher-Bolliger A., Reubi J.C., Maecke H.R. (2011). Alpha- versus beta-particle radiopeptide therapy in a human prostate cancer model (Bi-213-DOTA-PESIN and Bi-213-AMBA versus Lu-177-DOTA-PESIN). Cancer Res..

[44] Delle Fave G., Annibale B., de Magistris L., Severi C., Bruzzone R., Puoti M., Melchiorri P., Torsoli A., Erspamer V. (1985). Bombesin effects on human GI functions. Peptides.

[45] Bruzzone R., Tamburrano G., Lala A., Mauceri M., Annibale B., Severi C., de Magistris L., Leonetti F., Delle Fave G. (1983). Effect of bombesin on plasma insulin, pancreatic glucagon, and gut glucagon in man. J. Clin. Endocrinol. Metab..

[46] Rozengurt E. (1990). Bombesin stimulation of mitogenesis. Specific receptors, signal transduction, and early events. Am. Rev. Respir. Dis..

[47] Rozengurt E., Fabregat I., Coffer A., Gil J., Sinnett-Smith J. (1990). Mitogenic signalling through the bombesin receptor: Role of a guanine nucleotide regulatory protein. J. Cell Sci. Suppl..

[48] Stangelberger A., Schally A.V., Varga J.L., Zarandi M., Szepeshazi K., Armatis P., Halmos G. (2005). Inhibitory effect of antagonists of bombesin and growth hormone-releasing hormone on orthotopic and intraosseous growth and invasiveness of PC-3 human prostate cancer in nude mice. Clin. Cancer Res..

[49] Miyazaki M., Lamharzi N., Schally A.V., Halmos G., Szepeshazi K., Groot K., Cai R.Z. (1998). Inhibition of growth of MDA-MB-231 human breast cancer xenografts in nude mice by bombesin/gastrin-releasing peptide (GRP) antagonists RC-3940-II and RC-3095. Eur. J. Cancer.

[50] Ramos-Alvarez I., Moreno P., Mantey S.A., Nakamura T., Nuche-Berenguer B., Moody T.W., Coy D.H., Jensen R.T. (2015). Insights into bombesin receptors and ligands: Highlighting recent advances. Peptides.

[51] Maina T., Nock B.A., Kulkarni H., Singh A., Baum R.P. (2017). Theranostic prospects of gastrin-releasing peptide receptor-radioantagonists in oncology. PET Clin..

[52] Jensen R.T., Coy D.H. (1991). Progress in the development of potent bombesin receptor antagonists. Trends Pharmacol. Sci..

[53] de Castiglione R., Gozzini L. (1996). Bombesin receptor antagonists. Crit. Rev. Oncol. Hematol..

[54] Wang L.H., Coy D.H., Taylor J.E., Jiang N.Y., Moreau J.P., Huang S.C., Frucht H., Haffar B.M., Jensen R.T. (1990). *Des*-Met carboxyl-terminally modified analogues of bombesin function as potent bombesin receptor antagonists, partial agonists, or agonists. J. Biol. Chem..

[55] Nock B., Nikolopoulou A., Chiotellis E., Loudos G., Maintas D., Reubi J.C., Maina T. (2003). [^99m^Tc]Demobesin 1, a novel potent bombesin analogue for GRP receptor-targeted tumour imaging. Eur. J. Nucl. Med. Mol. Imaging.

[56] Cescato R., Maina T., Nock B., Nikolopoulou A., Charalambidis D., Piccand V., Reubi J.C. (2008). Bombesin receptor antagonists may be preferable to agonists for tumor targeting. J. Nucl. Med..

[57] Nock B.A., Charalambidis D., Sallegger W., Waser B., Mansi R., Nicolas G.P., Ketani E., Nikolopoulou A., Fani M., Reubi J.C. (2018). New gastrin releasing peptide receptor-directed [^99m^Tc]Demobesin 1 mimics: Synthesis and comparative evaluation. J. Med. Chem..

[58] Lymperis E., Kaloudi A., Sallegger W., Bakker I.L., Krenning E.P., de Jong M., Maina T., Nock B.A. (2018). Radiometal-dependent biological profile of the radiolabeled gastrin-releasing peptide receptor antagonist SB3 in cancer theranostics: Metabolic and biodistribution patterns defined by neprilysin. Bioconjug. Chem..

[59] Bakker I.L., van Tiel S.T., Haeck J., Doeswijk G.N., de Blois E., Segbers M., Maina T., Nock B.A., de Jong M., Dalm S.U. (2018). In vivo stabilized SB3, an attractive GRPR antagonist, for pre- and intra-operative imaging for prostate cancer. Mol. Imaging Biol..

[60] Llinares M., Devin C., Chaloin O., Azay J., Noel-Artis A.M., Bernad N., Fehrentz J.A., Martinez J. (1999). Syntheses and biological activities of potent bombesin receptor antagonists. J. Pept. Res..

[61] Mansi R., Wang X., Forrer F., Kneifel S., Tamma M.L., Waser B., Cescato R., Reubi J.C., Maecke H.R. (2009). Evaluation of a 1,4,7,10-tetraazacyclododecane-1,4,7,10-tetraacetic acid-conjugated bombesin-based radioantagonist for the labeling with single-photon emission computed tomography, positron emission tomography, and therapeutic radionuclides. Clin. Cancer Res..

[62] Gourni E., Mansi R., Jamous M., Waser B., Smerling C., Burian A., Buchegger F., Reubi J.C., Maecke H.R. (2014). N-terminal modifications improve the receptor affinity and pharmacokinetics of radiolabeled peptidic gastrin-releasing peptide receptor antagonists: Examples of ^68^Ga- and ^64^Cu-labeled peptides for PET imaging. J. Nucl. Med..

[63] Mansi R., Wang X., Forrer F., Waser B., Cescato R., Graham K., Borkowski S., Reubi J.C., Maecke H.R. (2011). Development of a potent DOTA-conjugated bombesin antagonist for targeting GRPR-positive tumours. Eur. J. Nucl. Med. Mol. Imaging.

[64] Marsouvanidis P.J., Nock B.A., Hajjaj B., Fehrentz J.A., Brunel L., M’Kadmi C., van der Graaf L., Krenning E.P., Maina T., Martinez J. (2013). Gastrin releasing peptide receptor-directed radioligands based on a bombesin antagonist: Synthesis, ^111^In-labeling, and preclinical profile. J. Med. Chem..

[65] Abiraj K., Mansi R., Tamma M.L., Forrer F., Cescato R., Reubi J.C., Akyel K.G., Maecke H.R. (2010). Tetraamine-derived bifunctional chelators for technetium-99m labelling: Synthesis, bioconjugation and evaluation as targeted SPECT imaging probes for GRP-receptor-positive tumours. Chemistry.

[66] Roivainen A., Kähkonen E., Luoto P., Borkowski S., Hofmann B., Jambor I., Lehtio K., Rantala T., Rottmann A., Sipila H. (2013). Plasma pharmacokinetics, whole-body distribution, metabolism, and radiation dosimetry of ^68^Ga bombesin antagonist BAY 86-7548 in healthy men. J. Nucl. Med..

[67] Varasteh Z., Rosenström U., Velikyan I., Mitran B., Altai M., Honarvar H., Rosestedt M., Lindeberg G., Sörensen J., Larhed M. (2014). The effect of mini-PEG-based spacer length on binding and pharmacokinetic properties of a ^68^Ga-labeled NOTA-conjugated antagonistic analog of bombesin. Molecules.

[68] Abiraj K., Mansi R., Tamma M.L., Fani M., Forrer F., Nicolas G., Cescato R., Reubi J.C., Maecke H.R. (2011). Bombesin antagonist-based radioligands for translational nuclear imaging of gastrin-releasing peptide receptor-positive tumors. J. Nucl. Med..

[69] Anderson C.J., Ferdani R. (2009). Copper-64 radiopharmaceuticals for PET imaging of cancer: Advances in preclinical and clinical research. Cancer Biother. Radiopharm..

[70] Dalm S.U., Bakker I.L., de Blois E., Doeswijk G.N., Konijnenberg M.W., Orlandi F., Barbato D., Tedesco M., Maina T., Nock B.A. (2017). ^68^Ga/^177^Lu-NeoBOMB1, a novel radiolabeled GRPR antagonist for theranostic use in oncology. J. Nucl. Med..

[71] Kaloudi A., Lymperis E., Giarika A., Dalm S., Orlandi F., Barbato D., Tedesco M., Maina T., de Jong M., Nock B.A. (2017). NeoBOMB1, a GRPR-antagonist for breast cancer theragnostics: First results of a preclinical study with [^67^Ga]NeoBOMB1 in T-47D cells and tumor-bearing mice. Molecules.

[72] Chatalic K.L., Kwekkeboom D.J., de Jong M. (2015). Radiopeptides for imaging and therapy: A radiant future. J. Nucl. Med..

[73] Vlieghe P., Lisowski V., Martinez J., Khrestchatisky M. (2010). Synthetic therapeutic peptides: Science and market. Drug. Discov. Today.

[74] Adessi C., Soto C. (2002). Converting a peptide into a drug: Strategies to improve stability and bioavailability. Curr. Med. Chem..

[75] Erak M., Bellmann-Sickert K., Els-Heindl S., Beck-Sickinger A.G. (2018). Peptide chemistry toolbox—Transforming natural peptides into peptide therapeutics. Bioorg. Med. Chem..

[76] Nock B.A., Maina T., Krenning E.P., de Jong M. (2014). “To serve and protect”: Enzyme inhibitors as radiopeptide escorts promote tumor targeting. J. Nucl. Med..

[77] Puente X.S., Sanchez L.M., Overall C.M., Lopez-Otin C. (2003). Human and mouse proteases: A comparative genomic approach. Nat. Rev. Genet..

[78] Shipp M.A., Tarr G.E., Chen C.Y., Switzer S.N., Hersh L.B., Stein H., Sunday M.E., Reinherz E.L. (1991). CD10/neutral endopeptidase 24.11 hydrolyzes bombesin-like peptides and regulates the growth of small cell carcinomas of the lung. Proc. Natl. Acad. Sci. USA.

[79] Linder K.E., Metcalfe E., Arunachalam T., Chen J., Eaton S.M., Feng W., Fan H., Raju N., Cagnolini A., Lantry L.E. (2009). In vitro and in vivo metabolism of Lu-AMBA, a GRP-receptor binding compound, and the synthesis and characterization of its metabolites. Bioconjug. Chem..

[80] Roques B.P. (1993). Zinc metallopeptidases: Active site structure and design of selective and mixed inhibitors: New approaches in the search for analgesics and anti-hypertensives. Biochem. Soc. Trans..

[81] Roques B.P., Noble F., Dauge V., Fournie-Zaluski M.C., Beaumont A. (1993). Neutral endopeptidase 24.11: Structure, inhibition, and experimental and clinical pharmacology. Pharmacol. Rev..

[82] Lymperis E., Kaloudi A., Kanellopoulos P., de Jong M., Krenning E.P., Nock B.A., Maina T. (2019). Comparing Gly^11^/dAla^11^-replacement vs. the in-situ neprilysin-inhibition approach on the tumor-targeting efficacy of the ^111^In-SB3/^111^In-SB4 radiotracer pair. Molecules.

[83] Maina-Nock T., Nock B.A., Laverman P., Marsouvanidis P.J., Krenning E.P., Boerman O., de Jong M. (2013). Dimerization is not always the best option for upgrading the in vivo profile of peptide radioligands. Eur. J. Nucl. Med. Mol. Imaging.

[84] Liolios C., Buchmuller B., Bauder-Wust U., Schafer M., Leotta K., Haberkorn U., Eder M., Kopka K. (2018). Monomeric and dimeric ^68^Ga-labeled bombesin analogues for positron emission tomography (PET) imaging of tumors expressing gastrin-releasing peptide receptors (GRPRs). J. Med. Chem..

[85] Lindner S., Michler C., Wangler B., Bartenstein P., Fischer G., Schirrmacher R., Wangler C. (2014). PESIN multimerization improves receptor avidities and in vivo tumor targeting properties to GRPR-overexpressing tumors. Bioconjug. Chem..

[86] Valverde I.E., Bauman A., Kluba C.A., Vomstein S., Walter M.A., Mindt T.L. (2013). 1,2,3-Triazoles as amide bond mimics: Triazole scan yields protease-resistant peptidomimetics for tumor targeting. Angew. Chem. Int. Ed..

[87] Valverde I.E., Vomstein S., Fischer C.A., Mascarin A., Mindt T.L. (2015). Probing the backbone function of tumor targeting peptides by an amide-to-triazole substitution strategy. J. Med. Chem..

[88] Maina T., Kaloudi A., Valverde I.E., Mindt T.L., Nock B.A. (2017). Amide-to-triazole switch vs. in vivo NEP-inhibition approaches to promote radiopeptide targeting of GRPR-positive tumors. Nucl. Med. Biol..

[89] Lymperis E., Kaloudi A., Kanellopoulos P., Krenning E.P., de Jong M., Maina T., Nock B.A. (2019). Comparative evaluation of the new GRPR-antagonist ^111^In-SB9 and ^111^In-AMBA in prostate cancer models: Implications of in vivo stability. J. Label. Compd. Radiopharm..

[90] Kanellopoulos P., Lymperis E., Kaloudi A., de Jong M., Krenning E.P., Nock B.A., Maina T. (2020). [^99m^Tc]Tc-DB1 mimics with different-length PEG spacers: Preclinical comparison in GRPR-positive models. Molecules.

[91] Kaloudi A., Lymperis E., Kanellopoulos P., Waser B., de Jong M., Krenning E.P., Reubi J.C., Nock B.A., Maina T. (2019). Localization of ^99m^Tc-GRP analogs in GRPR-expressing tumors: Effects of peptide length and neprilysin inhibition on biological responses. Pharmaceuticals.

[92] Popp I., Del Pozzo L., Waser B., Reubi J.C., Meyer P.T., Maecke H.R., Gourni E. (2017). Approaches to improve metabolic stability of a statine-based GRP receptor antagonist. Nucl. Med. Biol..

[93] Chatalic K.L., Konijnenberg M., Nonnekens J., de Blois E., Hoeben S., de Ridder C., Brunel L., Fehrentz J.A., Martinez J., van Gent D.C. (2016). In vivo stabilization of a gastrin-releasing peptide receptor antagonist enhances PET imaging and radionuclide therapy of prostate cancer in preclinical studies. Theranostics.

[94] Suda H., Aoyagi T., Takeuchi T., Umezawa H. (1973). Letter: A thermolysin inhibitor produced by actinomycetes: Phosphoramidon. J. Antibiot..

[95] Oefner C., D’Arcy A., Hennig M., Winkler F.K., Dale G.E. (2000). Structure of human neutral endopeptidase (neprilysin) complexed with phosphoramidon. J. Mol. Biol..

[96] Salazar-Lindo E., Santisteban-Ponce J., Chea-Woo E., Gutierrez M. (2000). Racecadotril in the treatment of acute watery diarrhea in children. N. Engl. J. Med..

[97] Roques B.P., Fournie-Zaluski M.C., Soroca E., Lecomte J.M., Malfroy B., Llorens C., Schwartz J.C. (1980). The enkephalinase inhibitor thiorphan shows antinociceptive activity in mice. Nature.

[98] Valkema R., Fröberg A., Maina T., Nock B.A., de Blois E., Melis M.L., Konijnenberg M.W., Koolen S.L.W., Peeters R.P., de Herder W.W. (2019). Clinical translation of the PepProtect: A novel method to improve the detection of cancer and metastases by peptide scanning under the protection of enzyme inhibitors. Eur. J. Nucl. Med. Mol. Imaging.

[99] Ayalasomayajula S., Langenickel T., Pal P., Boggarapu S., Sunkara G. (2017). Clinical pharmacokinetics of sacubitril/valsartan (LCZ696): A novel angiotensin receptor-neprilysin inhibitor. Clin. Pharmacokinet..

[100] McMurray J.J., Packer M., Solomon S.D. (2014). Neprilysin inhibition for heart failure. N. Engl. J. Med..

[101] Kanellopoulos P., Kaloudi A., Rouchota M., Loudos G., de Jong M., Krenning E.P., Nock B.A., Maina T. (2020). One step closer to clinical translation: Enhanced tumor targeting of [^99m^Tc]Tc-DB4 and [^111^In]In-SG4 in mice treated with Entresto. Pharmaceutics.

[102] Kulkarni H.R., Singh A., Langbein T., Schuchardt C., Mueller D., Zhang J., Lehmann C., Baum R.P. (2018). Theranostics of prostate cancer: From molecular imaging to precision molecular radiotherapy targeting the prostate specific membrane antigen. Br. J. Radiol..

[103] Minamimoto R., Hancock S., Schneider B., Chin F.T., Jamali M., Loening A., Vasanawala S., Gambhir S.S., Iagaru A. (2016). Pilot comparison of ^68^Ga-RM2 PET and ^68^Ga-PSMA-11 PET in patients with biochemically recurrent prostate cancer. J. Nucl. Med..

[104] Baratto L., Song H., Duan H., Hatami N., Bagshaw H., Buyyounouski M., Hancock S., Shah S.A., Srinivas S., Swift P. (2021). PSMA- and GRPR-targeted PET: Results from 50 patients with biochemically recurrent prostate cancer. J. Nucl. Med..

[105] Liolios C., Schafer M., Haberkorn U., Eder M., Kopka K. (2016). Novel bispecific PSMA/GRPR targeting radioligands with optimized pharmacokinetics for improved PET imaging of prostate cancer. Bioconjug. Chem..

[106] Mitran B., Varasteh Z., Abouzayed A., Rinne S.S., Puuvuori E., De Rosa M., Larhed M., Tolmachev V., Orlova A., Rosenström U. (2019). Bispecific GRPR-antagonistic anti-PSMA/GRPR heterodimer for PET and SPECT diagnostic imaging of prostate cancer. Cancers.

[107] Lundmark F., Abouzayed A., Mitran B., Rinne S.S., Varasteh Z., Larhed M., Tolmachev V., Rosenström U., Orlova A. (2020). Heterodimeric radiotracer targeting PSMA and GRPR for imaging of prostate cancer—Optimization of the affinity towards PSMA by linker modification in murine model. Pharmaceutics.

[108] Zhang J., Niu G., Lang L., Li F., Fan X., Yan X., Yao S., Yan W., Huo L., Chen L. (2017). Clinical translation of a dual integrin α_v_β_3_– and gastrin-releasing peptide receptor–targeting PET radiotracer, ^68^Ga-BBN-RGD. J. Nucl. Med..

[109] Zhang J., Mao F., Niu G., Peng L., Lang L., Li F., Ying H., Wu H., Pan B., Zhu Z. (2018). ^68^Ga-BBN-RGD PET/CT for GRPR and integrin alpha_v_beta_3_ imaging in patients with breast cancer. Theranostics.

[110] Mitran B., Rinne S.S., Konijnenberg M.W., Maina T., Nock B.A., Altai M., Vorobyeva A., Larhed M., Tolmachev V., de Jong M. (2019). Trastuzumab cotreatment improves survival of mice with PC-3 prostate cancer xenografts treated with the GRPR antagonist ^177^Lu-DOTAGA-PEG2 -RM26. Int. J. Cancer.

[111] Verhoeven M., Seimbille Y., Dalm S.U. (2019). Therapeutic applications of pretargeting. Pharmaceutics.

[112] D’Onofrio A., Silva F., Gano L., Karczmarczyk U., Mikolajczak R., Garnuszek P., Paulo A. (2021). Clickable radiocomplexes with trivalent radiometals for cancer theranostics: In vitro and in vivo studies. Front. Med..

[113] Verhoeven M.H., Chen K., de Jong M., Seimbille Y., Dalm S.U. (2020). Successful pretargeting approach for peptide receptor radionuclide therapy of GRPR-positive prostate cancer. Eur. J. Nucl. Med. Mol. Imaging.

[114] Dumont R.A., Tamma M., Braun F., Borkowski S., Reubi J.C., Maecke H., Weber W.A., Mansi R. (2013). Targeted radiotherapy of prostate cancer with a gastrin-releasing peptide receptor antagonist is effective as monotherapy and in combination with rapamycin. J. Nucl. Med..

[115] Grzmil M., Qin Y., Schleuniger C., Frank S., Imobersteg S., Blanc A., Spillmann M., Berger P., Schibli R., Béhé M. (2020). Pharmacological inhibition of mTORC1 increases CCKBR-specific tumor uptake of radiolabeled minigastrin analogue [^177^Lu]Lu-PP-F11N. Theranostics.

[116] Damiana T.S.T., Dalm S.U. (2021). Combination therapy, a promising approach to enhance the efficacy of radionuclide and targeted radionuclide therapy of prostate and breast cancer. Pharmaceutics.

[117] Zhang W., Fan W., Ottemann B.M., Alshehri S., Garrison J.C. (2020). Development of improved tumor-residualizing, GRPR-targeted agents: Preclinical comparison of an endolysosomal trapping approach in agonistic and antagonistic constructs. J. Nucl. Med..

[118] Müller C., Domnanich K.A., Umbricht C.A., van der Meulen N.P. (2018). Scandium and terbium radionuclides for radiotheranostics: Current state of development towards clinical application. Br. J. Radiol..

[119] Ferguson S., Wuest M., Richter S., Bergman C., Dufour J., Krys D., Simone J., Jans H.S., Riauka T., Wuest F. (2020). A comparative PET imaging study of ^44g^Sc- and ^68^Ga-labeled bombesin antagonist BBN2 derivatives in breast and prostate cancer models. Nucl. Med. Biol..

[120] Morgenstern A., Apostolidis C., Kratochwil C., Sathekge M., Krolicki L., Bruchertseifer F. (2018). An overview of targeted alpha therapy with (225) actinium and (213) bismuth. Curr. Radiopharm..

[121] de Koning H.J., Gulati R., Moss S.M., Hugosson J., Pinsky P.F., Berg C.D., Auvinen A., Andriole G.L., Roobol M.J., Crawford E.D. (2018). The efficacy of prostate-specific antigen screening: Impact of key components in the erspc and plco trials. Cancer.

[122] Drost F.H., Osses D., Nieboer D., Bangma C.H., Steyerberg E.W., Roobol M.J., Schoots I.G. (2020). Prostate magnetic resonance imaging, with or without magnetic resonance imaging-targeted biopsy, and systematic biopsy for detecting prostate cancer: A COCHRANE systematic review and meta-analysis. Eur. Urol..

[123] Luiting H.B., van Leeuwen P.J., Busstra M.B., Brabander T., van der Poel H.G., Donswijk M.L., Vis A.N., Emmett L., Stricker P.D., Roobol M.J. (2020). Use of gallium-68 prostate-specific membrane antigen positron-emission tomography for detecting lymph node metastases in primary and recurrent prostate cancer and location of recurrence after radical prostatectomy: An overview of the current literature. BJU Int..

[124] Van de Wiele C., Sathekge M., de Spiegeleer B., De Jonghe P.J., Debruyne P.R., Borms M., Beels L., Maes A. (2020). PSMA expression on neovasculature of solid tumors. Histol. Histopathol..

[125] Virgolini I., Decristoforo C., Haug A., Fanti S., Uprimny C. (2018). Current status of theranostics in prostate cancer. Eur. J. Nucl. Med. Mol. Imaging.

[126] Dorff T.B., Fanti S., Farolfi A., Reiter R.E., Sadun T.Y., Sartor O. (2019). The evolving role of prostate-specific membrane antigen-based diagnostics and therapeutics in prostate cancer. Am. Soc. Clin. Oncol. Educ. Book.

[127] Crocerossa F., Marchioni M., Novara G., Carbonara U., Ferro M., Russo G.I., Porpiglia F., Di Nicola M., Damiano R., Autorino R. (2021). Detection rate of prostate specific membrane antigen tracers for positron emission tomography/computerized tomography in prostate cancer biochemical recurrence: A systematic review and network meta-analysis. J. Urol..

[128] Nuhn P., De Bono J.S., Fizazi K., Freedland S.J., Grilli M., Kantoff P.W., Sonpavde G., Sternberg C.N., Yegnasubramanian S., Antonarakis E.S. (2019). Update on systemic prostate cancer therapies: Management of metastatic castration-resistant prostate cancer in the era of precision oncology. Eur. Urol..

[129] Ahmadzadehfar H., Rahbar K., Baum R.P., Seifert R., Kessel K., Bogemann M., Kulkarni H.R., Zhang J., Gerke C., Fimmers R. (2021). Prior therapies as prognostic factors of overall survival in metastatic castration-resistant prostate cancer patients treated with [^177^Lu]Lu-PSMA-617. A WARMTH multicenter study (the 617 trial). Eur. J. Nucl. Med. Mol. Imaging.

[130] Zhang J., Singh A., Kulkarni H.R., Schuchardt C., Müller D., Wester H.J., Maina T., Rösch F., van der Meulen N.P., Müller C. (2019). From bench to bedside-the Bad Berka experience with first-in-human studies. Semin. Nucl. Med..

[131] Barber T.W., Singh A., Kulkarni H.R., Niepsch K., Billah B., Baum R.P. (2019). Clinical outcomes of ^177^Lu-PSMA radioligand therapy in earlier and later phases of metastatic castration-resistant prostate cancer grouped by previous taxane chemotherapy. J. Nucl. Med..

[132] von Eyben F.E., Roviello G., Kiljunen T., Uprimny C., Virgolini I., Kairemo K., Joensuu T. (2018). Third-line treatment and ^177^Lu-PSMA radioligand therapy of metastatic castration-resistant prostate cancer: A systematic review. Eur. J. Nucl. Med. Mol. Imaging.

[133] Khreish F., Ghazal Z., Marlowe R.J., Rosar F., Sabet A., Maus S., Linxweiler J., Bartholoma M., Ezziddin S. (2021). ^177^Lu-PSMA-617 radioligand therapy of metastatic castration-resistant prostate cancer: Initial 254-patient results from a prospective registry (reality study). Eur. J. Nucl. Med. Mol. Imaging.

[134] Feuerecker B., Tauber R., Knorr K., Heck M., Beheshti A., Seidl C., Bruchertseifer F., Pickhard A., Gafita A., Kratochwil C. (2021). Activity and adverse events of actinium-225-PSMA-617 in advanced metastatic castration-resistant prostate cancer after failure of lutetium-177-PSMA. Eur. Urol..

[135] Gnesin S., Cicone F., Mitsakis P., Van der Gucht A., Baechler S., Miralbell R., Garibotto V., Zilli T., Prior J.O. (2018). First in-human radiation dosimetry of the gastrin-releasing peptide (GRP) receptor antagonist ^68^Ga-NODAGA-MJ9. EJNMMI Res..

[136] Ballal S., Yadav M.P., Sahoo R.K., Tripathi M., Dwivedi S.N., Bal C. (2021). ^225^Ac-PSMA-617-targeted alpha therapy for the treatment of metastatic castration-resistant prostate cancer: A systematic review and meta-analysis. Prostate.

[137] Bandari R.P., Carmack T.L., Malhotra A., Watkinson L., Fergason Cantrell E.A., Lewis M.R., Smith C.J. (2021). Development of heterobivalent theranostic probes having high affinity/selectivity for the GRPR/PSMA. J. Med. Chem..

